# A case of autosomal dominant polycystic kidney disease with amelioration of refractory cyst infections following prolonged hemodialysis time

**DOI:** 10.1007/s13730-021-00614-w

**Published:** 2021-06-17

**Authors:** Norio Ieiri, Osamu Hotta

**Affiliations:** 1 Division of Internal Medicine, Hotta Osamu Clinic, Rokuchounomeminamimach 2-39, Wakabayashi-ku, Sendai, Miyagi 984-0013 Japan; 2 Division of Nephrology and Endocrinology, Tohoku Medical and Pharmaceutical University, Sendai, Japan

**Keywords:** Autosomal dominant polycystic kidney disease (ADPKD), Renal cyst infection, 8-h Thrice weekly nocturnal hemodialysis, C-reactive protein, Neutrophil/lymphocyte ratio (NLR), Hemodialysis product (HDP)

## Abstract

Renal cyst infection is a frequent and serious problem in patients with autosomal dominant polycystic kidney disease (ADPKD). Cyst infection is often a refractory complication of treatment that leads to sepsis and death in patients with ADPKD. It was previously reported that a higher dose of dialysis demonstrated clearly better survival than shorten-time dialysis. The relationship between the frequency of cyst infection episodes in hemodialysis (HD) patients with ADPKD and the dialysis dose has not yet been fully elucidated. In this report, we describe a case of an HD patient with ADPKD that was provided elongation of HD time from 4-h twice weekly HD to 8-h thrice weekly nocturnal HD. As a result, the frequency of cyst infection episodes decreased from 10.0 to 1.5 days a month. Our findings suggest that prolonged HD time might contribute to amelioration of refractory cyst infections in patients with ADPKD.

## Introduction

Autosomal dominant polycystic kidney disease (ADPKD) is the most common inherited kidney disease that accounts for approximately 10% of all patients with end-stage renal disease in Japan. ADPKD is characterized by massive enlargement of the kidneys as a result of progressive expansion of multiple bilateral renal cysts. Patients with ADPKD have the following serious problems: cyst infection leading to mortality [1] and intra-abdominal pressure resulting in poor oral intake and consequent malnutrition [2]. Inflammation is a risk factor for increased cardiovascular mortality in hemodialysis (HD) patients [3] with ADPKD and preserved kidney function; it increases with worse renal function [4]. In one study, higher dose of dialysis resulted in clearly better survival than shorter dialysis times [5].

In this report, we describe the first case of an HD patient with ADPKD that resulted in a decrease in cyst infection episodes, decrease levels of inflammatory markers, and improvement of nutritional status following prolonged HD time.

## Case report

A 46-year-old Japanese man with a history of distal gastrectomy for gastric cancer visited our hospital on November 2011, because he wanted a more adequate dose of HD than the dialysis schedule (four hours twice a week) he was receiving at his facility at that time. The primary cause of his renal failure was ADPKD, and he started HD in October 2009. His dialysis schedule was 3 h twice a week until October 2011, when he suffered palpitations and shortness of breath due to congestive heart failure. His mother and young sister suffered from end-stage renal disease due to ADPKD. His height and weight were 175 cm and 72.1 kg, respectively, at the first visit to our hospital. His blood pressure was 201/122 mmHg. On physical examination, he had severe leg edema. Chest radiography revealed a high cardiothoracic ratio (55.9%), and his electrocardiogram showed no remarkable change. Extracellular fluid was gradually removed by ultrafiltration during his 5-h thrice weekly HD just after he started dialysis at our hospital on November 9, 2011. He subsequently recovered from this volume overload state. Four months after starting treatment, he became normotensive and had a lower cardiothoracic ratio.

**Fig. 1:**
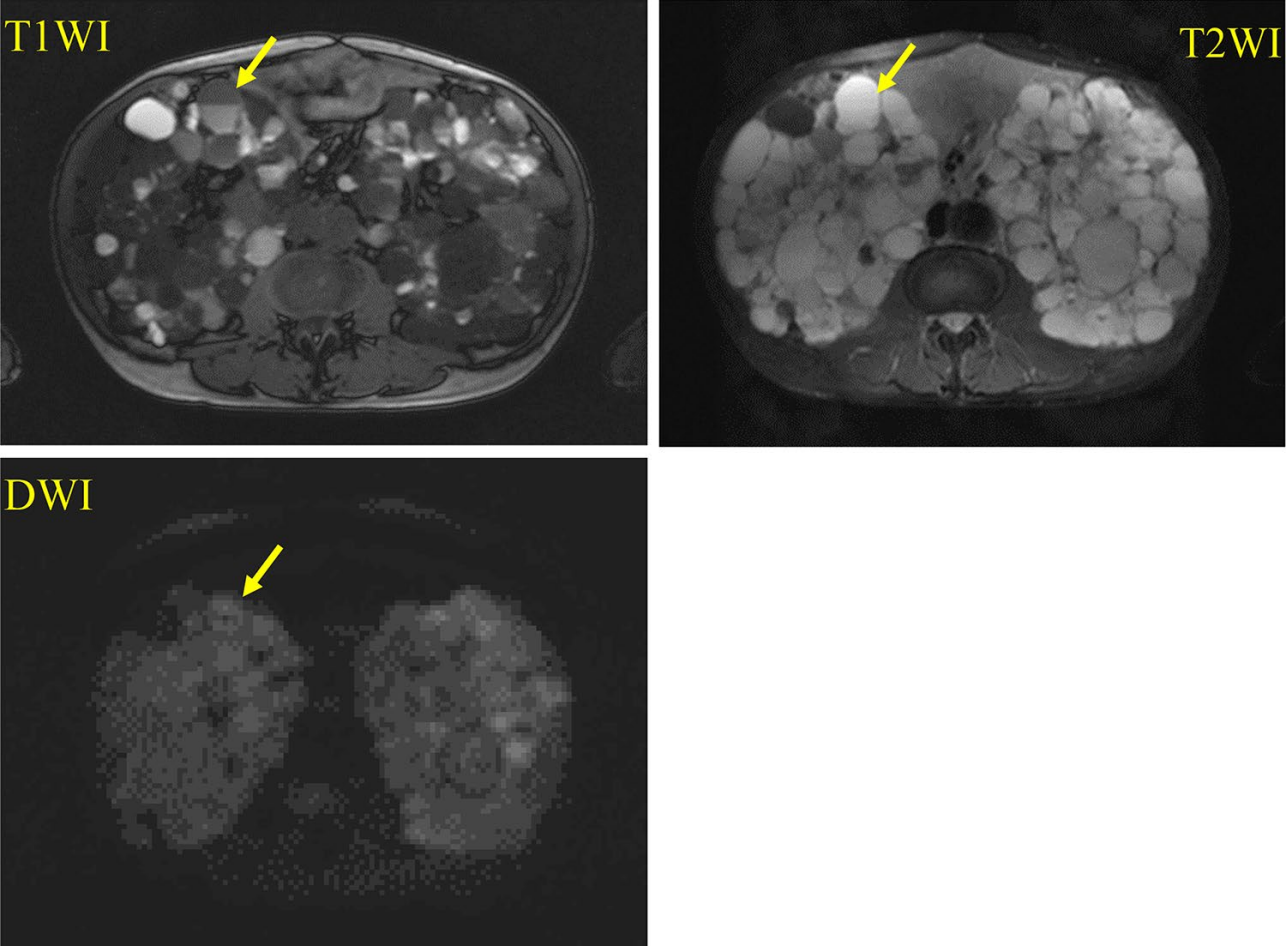
Abdominal MRI findings (T1WI, T2WI, and DWI). The infected renal cyst showed higher intensity on T1WI, T2WI, and DWI compared with normal cysts, and had a fluid–fluid level

**Fig. 2:**
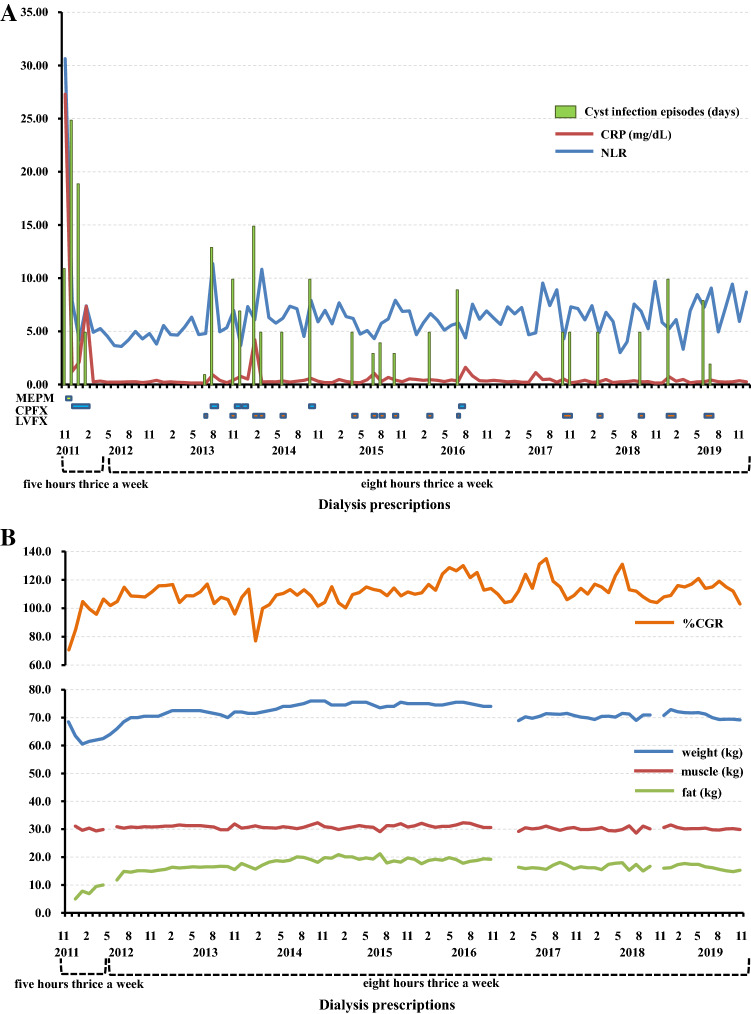
Summary of clinical course in terms of **A** inflammatory markers and **B** nutritional status. He suffered from cyst infection with critical serum CRP levels and high NLR soon after he started dialysis at our hospital. Moderate levels of inflammatory markers continued during his conventional HD period. Following the initiation of 8-h thrice weekly nocturnal hemodialysis on May 2012, the frequency of cyst infection episodes decreased, and there was stabilization of levels of inflammatory markers and improvement and maintenance of %CGR and fat mass. Since he fell and suffered a severe laceration of his tongue and fracture on his mandible and ribs on January 2017, his weight and fat mass decreased because of difficulty taking meals and reduction in his movement. *MEPM* meropenem hydrate, *CPFX* ciprofloxacin hydrochloride, *LVFX* levofloxacin hydrate

He suffered from high fever and abdominal pain with serum C-reactive protein (CRP) 27.29 mg/dL soon after he started dialysis at our hospital. Abdominal magnetic resonance imaging (MRI) revealed an infected renal cyst with higher intensity on T1-weighted image (T1WI), T2-weighted image (T2WI), and diffusion-weighted image (DWI) compared with normal cysts, and that had a fluid–fluid level (Fig. 1). The type of causative organism was not confirmed, because blood cultures obtained from him were negative. He was administered meropenem hydrate for 9 days followed by ciprofloxacin hydrochloride (CPFX) for 7 days on admission. He needed to receive CPFX for about 3 months after he was discharged from the hospital because of fever and abdominal pain with moderate serum CRP levels. The frequency of cyst infection episodes with fever, abdominal pain, and moderate serum CRP level were 10.0 days a month during his conventional HD period (November 2011 to April 2012). Following the initiation of 8-h thrice weekly nocturnal HD on May 2012 and until December 2019, the frequency of cyst infection episodes decreased to 1.5 days a month. Moreover, he attained a decrease and stabilization in levels of inflammatory markers and an improvement and maintenance of nutritional status compared with the conventional HD period (Fig. 2A, B).

## Discussion

This report describes the first case of an HD patient with ADPKD who experienced a decreased number of cyst infection episodes, decreased levels of inflammatory markers, and improvement of nutritional status in the context of prolonged HD time.

**Fig. 3:**
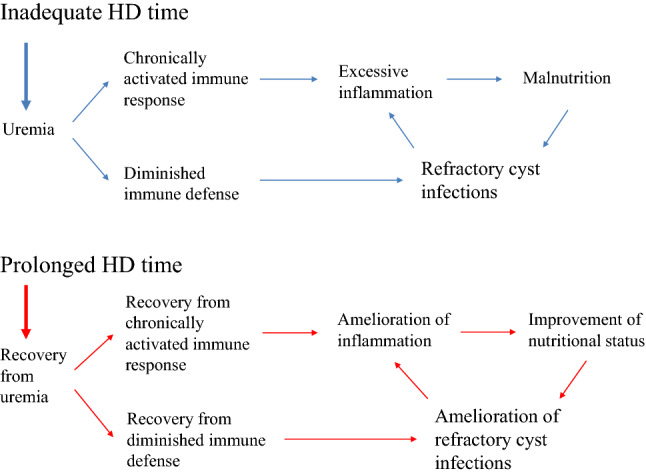
The schema of our case shows that uremia caused by insufficient dose of HD contributing to severe infection and malnutrition via disturbances of the immune system. Recovery from uremia following prolonged HD time contributing to amelioration of refractory cyst infections and malnutrition with improvement of immune system function

**Fig. 4:**
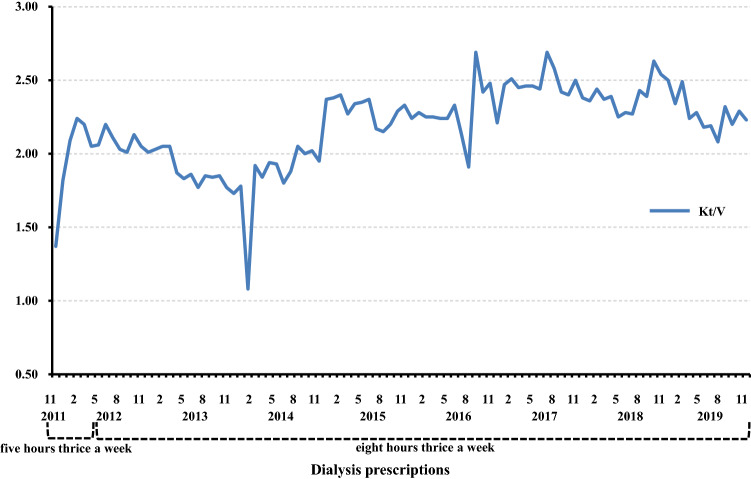
The changes in Kt/V of our case. The values of Kt/V increased soon after he started dialysis at our hospital and maintained more adequate degree after the initiation of 8-h thrice weekly nocturnal HD except for February 2014, when he was forced to be dialyzed shorter time because of appearance of Influenza A symptom

Renal cyst infection, a frequent complication of ADPKD, is often difficult to treat and consequently leads to death [1]. Lipid-permeable anti-microbial agents such as fluoroquinolones are the standard treatment for cyst infections [6]. However, cyst infection may recur even after adequate periods of antibiotic therapy [6]. Indeed, our patient needed to continue antibiotic administration for about 3 months after left the hospital because of fever and abdominal pain with moderate serum CRP levels. Because of variable comorbid conditions, causative pathogens, and other clinical factors in patients with ADPKD with renal cyst infection, it is difficult to develop an effective approach for the management of those patients [7]. In the context of uremia, moreover, diminished immune defenses with depletion of dendritic cells, naïve and central memory T cells, and B cells contribute to the high prevalence of infections and disturbance of polymorphonuclear leukocyte functions particularly give rise to increased risk for bacterial infections [8, 9] (Fig. 3). Our patient was assumed to be uremic at the first visit to our hospital based on inadequate hemodialysis product (HDP), calculated by (hours per HD session) × (HD sessions per week)^2^, as proposed by Scribner and Oreopoulos [10]. We believe our patient recovered from uremia based on the increase of HDP (from 16 to 72) and the improvement of Kt/V [11] (Fig. 4) as an index of HD adequacy, as well as the subsequent improvement in disturbances of the immune system and the reduction in cyst infections (Fig. 3).

Inflammation exists in ADPKD with preserved kidney function and increases with worse renal function [4]. Inflammatory markers are powerful predictors of cardiovascular risk and mortality in HD patients [3]. The prevention and treatment of inflammation is a high priority in patients on HD [12]. HD techniques such as nocturnal HD that result in greater removal of toxins contribute to the elimination of proinflammatory substances [12]. Our patient achieved lower and stabilized levels of inflammatory markers following the initiation of 8-h thrice weekly nocturnal HD. Prolonged HD time might be useful for the prevention of inflammation (Fig. 3). We used CRP and neutrophil/lymphocyte ratio (NLR) as inflammatory markers. CRP is the gold standard among the microinflammatory markers in HD [3] and has become a routine test in HD units to signal inflammation [12]. The NLR is easily calculated in peripheral blood and has been found to be a valuable index for predicting adverse clinical outcomes and estimating survival in various clinical settings such as cardiology practices [13].

The mass effect of the enlarged kidneys and liver in patients with ADPKD was significantly associated with malnutrition [2]. Furthermore, gastric resection, whether partial or total gastrectomy, often results in nutrition-related complications including weight loss [14]. Regardless of unfavorable nutritional conditions such as enlarged kidneys caused by ADPKD or post-distal gastrectomy state, our patient showed an increased percent creatinine generation rate (%CGR), developed by Shinzato et al. [15], and fat mass.

Several studies showed that protein–energy malnutrition increases mortality as well as the risk of cardiovascular disease [16]. Muscle mass is useful for estimating protein nutritional status [17]. One study showed that muscle mass, measured using computed tomography, significantly correlated with creatinine production in HD patients [18]. This suggests that the increase in the %CGR in our case indicated an improvement in protein nutritional status.

Obesity is a conventional risk factor for cardiovascular morbidity and mortality in the general population. Recently, a large number of studies reported an “obesity paradox,” i.e., higher body mass index, was paradoxically associated with better survival in HD patients [19]. An observational cohort study of 808 Japanese HD patients showed that increased fat mass was independently associated with low risk of non-cardiovascular death [20]. We believe that the increased fat mass in our patient gave a better nutritional outcome. HD patients with malnutrition have excessive inflammation with a chronically activated immune systems caused by many adverse conditions, including uremia [9] (Fig. 3). Therefore, it is possible that our patient’s improvement in nutritional status was derived from the amelioration of excessive inflammation with chronically activated immune system caused by uremia following prolonged HD time (Fig. 3).

It is the limitation of this report that only a single case we described was insufficient to show the causality between the amelioration of refractory cyst infections and prolonged HD time. Moreover, there is a possibility that the amelioration of refractory cyst infections was attributed to more effective treatment with antimicrobials in addition to prolonged HD time. We used mainly levofloxacin hydrate (LVFX) after prolonged HD time, whereas CPFX before that (Fig. 2A). The report of case series is desirable to suggest the direct causal relationship between the amelioration of refractory cyst infections and prolonged HD time. However, it is difficult to present case series with similar episodes because of various prescriptions of HD. We conducted a longer term observation study for the present case.
